# Indolent Mastocytosis and Bone Health: Molecular Mechanisms and Emerging Treatment Options

**DOI:** 10.3390/ijms26125649

**Published:** 2025-06-12

**Authors:** Lucia Jankovski, Matej Rakusa, Andrijana Koceva, Andrej Janež, Peter Kopač, Mojca Jensterle

**Affiliations:** 1 Faculty of Medicine, University of Ljubljana, 1000 Ljubljana, Slovenia; 2 Department of Endocrinology, Diabetes and Metabolic Diseases, University Medical Center Ljubljana, 1000 Ljubljana, Slovenia; 3 Department of Endocrinology and Diabetology, University Medical Center Maribor, 2000 Maribor, Slovenia; 4 Faculty of Medicine, University of Maribor, 2000 Maribor, Slovenia; 5 Allergology Department, University Hospital of Respiratory and Allergic Diseases Golnik, 4204 Golnik, Slovenia

**Keywords:** systemic mastocytosis, indolent systemic mastocytosis, bone health, osteoporosis, osteosclerosis

## Abstract

Mastocytosis is a heterogeneous group of disorders, distinguished by the monoclonal proliferation of mast cells (MCs) in one or more organs. While cutaneous mastocytosis (CM) is restricted to the skin, systemic mastocytosis (SM) presents with high MC infiltration of various organs. Indolent systemic mastocytosis (ISM) is the most common form in individuals with adult-onset of the disease. Bone health impairment is present in the vast majority of patients, ranging from osteoporosis to osteosclerosis, often presenting with fragility fractures. In this review, we comprehensively examine the impact of ISM on bone health, with particular emphasis on the molecular and cellular mechanisms underlying skeletal involvement, the clinical heterogeneity of bone manifestations, and the limitations of current diagnostic tools, while also evaluating emerging therapeutic strategies that target both MC activity and bone remodeling pathways.

## 1. Introduction

**Figure 1:**
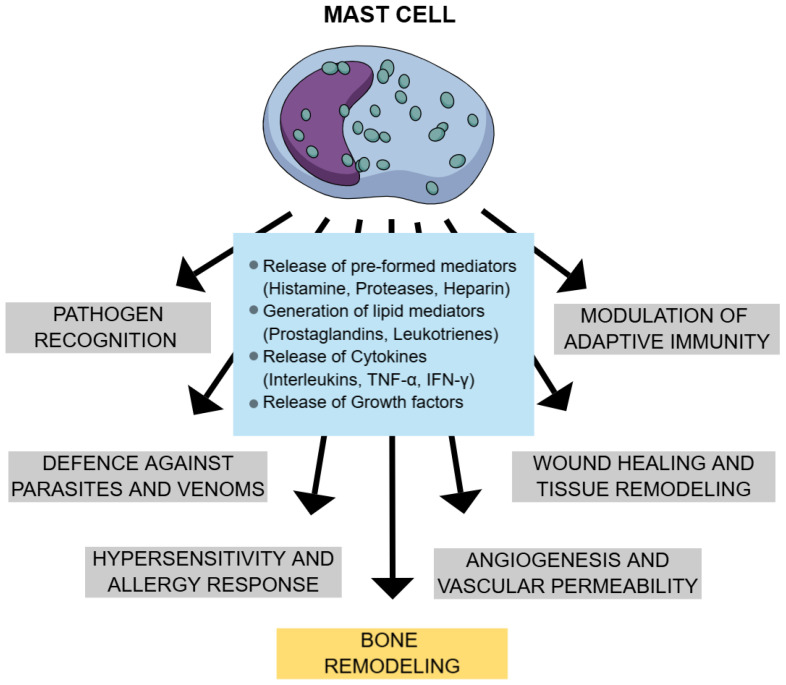
Physiological effects of mast cells. Legend: TNF α—tumor necrosis factor alpha; IFN-γ—interferon-gamma.

Mast cells (MCs) are specialized immune cells that are recognized for their role in maintaining immune homeostasis and are central to the pathogenesis of several diseases, including mastocytosis. These cells originate from multipotential stem cells in the bone marrow and yolk sac. Immature mast cells circulate throughout the body and migrate into tissues, where they differentiate into functional forms and reside [1,2,3]. Mast cells express diverse surface and cytoplasmic receptors, such as FcεRI, KIT, mas-related G protein-coupled receptor X2 (MRGPRX2), Toll-like receptors (TLRs), and complement receptors, that enable them to detect and respond to immunologic and non-immunologic stimuli. Activation may occur via IgE-mediated FcεRI crosslinking or through direct stimulation by microbes, drugs, enzymes, or physical factors. Upon activation, mast cells release pre-formed mediators (e.g., histamine, proteases, heparin) via degranulation and generate lipid mediators (e.g., prostaglandins, leukotrienes) and cytokines de novo [2,4,5]. These mediators contribute to inflammation, tissue remodeling, angiogenesis, and immune regulation [4,6,7]. Their adaptability and signaling abilities make mast cells crucial defenders and regulators in healthy physiology. They support both innate and adaptive immunity, help control infections and regulate inflammation. In the gut, they aid microbiome balance by influencing IgA and T and B cell maturation [8]. Mast cells contribute to host defense by recognizing and responding to a broad spectrum of pathogens, including bacteria, parasites, and fungi [4]. They mediate antimicrobial activity through the release of bioactive mediators, including antimicrobial peptides. Additionally, mast cell-derived proteases, including tryptase and chymase, facilitate the degradation and inactivation of exogenous toxins, such as those found in insect and snake venoms [4,9]. Mast cells are also essential for wound healing—guiding inflammation, tissue repair, and angiogenesis [10]. In blood vessels, they mediate vasodilation and vascular remodeling. Additionally, they support bone remodeling and mineral balance [11]. Figure 1 summarizes the physiological actions of mast cells.

Mastocytosis is a rare group of disorders, distinguished by the aberrant malignant proliferation and accumulation of atypical mast cells in various tissues, with skin and bone marrow (BM) being the most commonly affected [12,13]. The primary reason mastocytosis occurs is the overgrowth and activation of mast cells, predominantly driven by gain-of-function mutations in the KIT receptor. KIT is described as a master regulator of the mast cell lineage. Mutations in *KIT*, most often the *KIT* p.D816V activating point mutation, lead to uncontrolled mast cell growth and activity. This results in an abnormal increase in the mast cell burden in various organs and tissues [14,15]. Its diagnosis and categorization rely on criteria delineated in the 2022 International Consensus Classification (ICC) of Myeloid Neoplasms and Acute Leukemias and the 5th edition of the WHO Classification of Hematolymphoid Tumors [16]. A main criterion and four minor criteria have been established for the diagnosis of systemic mastocytosis (SM). It is diagnosed upon the fulfillment of one major criterion and one minor criterion, or three minor criteria. The major SM diagnostic criterion is the existence of multifocal compact MC infiltrates in clusters (≥15 MCs per cluster) in the BM or in other extracutaneous organs [17]. It is highly advisable to perform immunohistochemical staining utilizing antibodies targeting KIT and tryptase [17]. Minor criteria can also be used: the first one is the abnormal MC morphology (>25% of MCs), characterized by spindle-shaped MCs, atypical granulation, or cytoplasmic projections. Another minor criterion is the anomalous MC expression of CD25 and/or CD2 antigens, as these are expressed only on neoplastic MCs in SM and not on normal or reactive MCs [17]. Furthermore, flow cytometry is used to detect the expression of aberrant MC markers [17]. Additionally, the majority of patients with SM exhibit an activating point mutation at codon 816, particularly *KIT* D816V [17]. Lastly, the serological detection of a persistently elevated baseline serum tryptase level (BST) (>20 ng mL) is employed as a minor criterion [17].

The clinical features of mastocytosis vary widely. In children, it often presents as cutaneous mastocytosis (CM), limited to the skin, with symptoms typically beginning within the first two years of life and often resolving spontaneously by puberty [17]. More advanced forms, classified as systemic mastocytosis (SM), involve at least one extracutaneous organ—most commonly the spleen, lymph nodes, gastrointestinal tract, or bones [17]. Individuals with advanced forms of the disease (AdvSM) can experience organ damage due to the malignant proliferation and accumulation of neoplastic MCs. Furthermore, depending on the specific subtype, SM can lead to multi-organ dysfunction, and a life expectancy that can vary from just a few months to several years [18,19].

The majority of adult patients exhibit indolent systemic mastocytosis (ISM), which is primarily defined by symptoms related to MC mediators, with or without cutaneous manifestations, the absence of organ dysfunction, and a life expectancy that is nearly normal [17]. Bone marrow mastocytosis (BMM) is a clinicopathologic form of ISM marked by mild bone marrow involvement, absence of skin lesions, normal or mildly raised tryptase levels, and often linked to severe allergic reactions to Hymenoptera stings [20]. Currently, there are no epidemiological studies available that establish the exact incidence, point prevalence, or cumulative prevalence of ISM within the general population; nevertheless, the estimated cumulative prevalence of ISM by various studies is roughly 1 in 10,000 individuals [21].

ISM is recognized as a complex condition that can present with a broad spectrum of MC-mediated symptoms, including flushing, pruritus, dizziness, fainting, hypotensive shock, abdominal discomfort, nausea, vomiting, diarrhea, fatigue, depression, cognitive disturbances, palpitations, rapid heartbeat, and breathing difficulties, as well as bone or joint pain, muscle aches, and skeletal impairment [22]. These widespread signs are believed to result from the release of histamine, heparin, prostaglandins, chemokines, cytokines, neutral proteases, and acid hydrolases by MCs [23]. The symptoms and signs of ISM may be disregarded or deemed trivial by healthcare professionals lacking familiarity with the condition, which often leads to diagnostic delay [24]. A significant subset of patients comprises those with severe Hymenoptera venom allergy (HVA), where anaphylaxis could be the first clinical sign of mast cell disease. The prevalence of SM in individuals with HVA has been reported to range from 1% to 7.9%, markedly higher than in the general adult population [25,26,27]. Recent data indicate a greater-than-anticipated burden of clonal mast cell disorders among patients with severe HVA. The *KIT* D816V mutation in peripheral blood was identified in 21% of these patients; among them, 63% were diagnosed with BMM and 12% with ISM.

Importantly, only 15% of patients with detectable KIT D816V exhibited BST levels above 20 ng/mL [28]. These findings highlight the limited sensitivity of BST in detecting underlying SM in the context of HVA and underscore the importance of genetic testing in patients with a high clinical suspicion of clonal mast cell disease. The aim of this review is to comprehensively examine the impact of ISM on bone health, with particular emphasis on the molecular and cellular mechanisms underlying skeletal involvement, the clinical heterogeneity of bone manifestations, and the limitations of current diagnostic tools. Given that bone abnormalities are frequently observed in ISM—even in the absence of systemic symptoms—this review also evaluates emerging therapeutic strategies that target both MC activity and bone remodeling pathways. By integrating available clinical and experimental data, we seek to highlight the importance of early recognition and targeted management of bone disease in ISM to prevent complications, improve quality of life, and guide future research directions.

## 2. Manifestations of Bone Health Impairment

The involvement of bone in SM presents a compelling clinical model, attributed to the proximity of MCs to bone remodeling sites and their production of several chemical mediators and cytokines that might influence bone turnover [17]. Bone health impairment in SM is heterogeneous, ranging from osteoporosis, low bone mineral density (BMD), osteolysis, osteosclerosis, and bone marrow infiltrates, often presenting with mixed patterns as well [29]. Bone health impairment occurs in 50–70% of patients with SM [30,31]. The prevalence and subtype of bone impairment differ among distinct subtypes of SM [32].

Data from a German cohort of 1374 patients referred for bone biopsy due to osteoporosis revealed a 0.5% prevalence of ISM, with the rate rising to over 5% among young males with osteoporosis [33]. Furthermore, findings from a large cohort study involving 300 ISM patients indicated that the incidence of ISM peaks between the fourth and sixth decades of life [34].

Osteoporosis is the most common skeletal manifestation, with a prevalence of up to 60% in SM [31,35]. In patients with ISM, the prevalence of osteoporosis—defined as a lumbar spine or hip BMD T-score of ≤−2.5 standard deviations (SD)—is estimated to range between 20% and 38% [36,37,38,39,40]. In addition, ISM-induced low BMD—defined as a lumbar spine or hip BMD Z-score of ≤−2.0 SD—is reported in approximately 20% of patients, with a higher prevalence in males [37]. Osteopenia, a milder reduction in bone density that precedes osteoporosis, has also been observed in 32% of ISM patients [27].

Bone histomorphometric analysis in patients with ISM-induced osteoporosis showed significantly reduced trabecular bone volume, thickness, and number, with an increase in osteoblast and osteoclast number. Higher osteoclast indices were found in patients displaying MC granulomas compared to patients with diffuse mass cell distribution, suggesting that the bone impairment in ISM may depend on the type of mast cell distribution [36]. Osteoporosis is also more commonly detected in the lumbar spine than in the hip, with 20% of male patients with ISM showing medial collapses of vertebral bodies that resemble those commonly observed in glucocorticoid-induced osteoporosis [41]. All these findings indicate a significant involvement of trabecular over cortical bone. The reasons for this selective engagement remain ambiguous, probably due to the increased likelihood of clonal MCs colonizing the BM or the rapid rate of bone loss, which frequently involves the most metabolically active bone tissue [17,41]. Furthermore, it is possible that the influence of MC mediators on bone metabolism and the secretion of these mediators differ between sexes, as men with ISM are more susceptible to osteoporotic involvement compared to women [37,39].

Osteosclerosis, on the other hand, affects approximately 5–6% of patients with ISM [32,36], with a higher prevalence in more advanced subtypes of SM [32]. Bone histomorphometric analysis of ISM-induced osteosclerosis showed significantly increased trabecular bone volume and trabecular number, increased osteoclast and osteoblast number, decreased mineral content, and increased heterogeneity of the calcium distribution. This indicated a high bone turnover state, which results in osteosclerotic, poorly mineralized bone. ISM-induced osteosclerosis appears to be more common in females and presents with increased BMD and increased alkaline phosphate levels [36].

Bone impairment in ISM may be asymptomatic or may present with pain, potentially due to microscopic stress fractures [24]. In fact, bone pain is one of the hallmarks of mastocytosis-related osteoporosis, which can be debilitating, especially in the case of extensive bone marrow infiltration [17]. The pain is frequently located in long bones, such as the femur and pelvic bones; occasionally, it can involve joints and smaller bones, including the skull, spine, ribs, and hands [42]. According to a case-control cohort study by Hermine et al., patients commonly (54%) experienced poorly localized bone pain, which was, in 18% of instances, classified as severe or intolerable [23].

In asymptomatic cases, without a history of anaphylaxis, idiopathic osteoporosis, or an unexplained fragility fracture, may be the only indication of a latent ISM [17]. The age- and sex-related prevalence of fragility fractures was evaluated by van der Veer et al. in a study including 157 patients diagnosed with ISM [39]. Among patients younger than 50 years, osteoporotic fractures were reported in 35% of men and 6% of women. In contrast, among older patients, the prevalence increased significantly, with 62% of men and 40% of women affected [39]. Overall, 27% of patients had one or more vertebral fractures, with a mean of 2.1 fractures per patient, while 21% sustained non-vertebral fractures, with a mean of 1.6 per patient [39]. In this group, bone impairment (osteoporosis, osteosclerosis, or osteoporotic fracture) was the first presenting symptom in 18 of the patients [39].

Fragility fractures occur in 33% to 43% of patients with SM [35,40] and in 24% to 41% of patients with ISM [36,39,43]. These fractures are frequently multiple [33,40] and predominantly affect the vertebral bodies, while the peripheral skeleton is affected less commonly [35].

Various predictors of fragility fracture risk have been evaluated. Studies have shown that there is a higher prevalence of fragility fractures in patients with ISM without cutaneous manifestations compared to patients with skin lesions [36,39], although this has not always been confirmed [37]. Age at diagnosis, low hip BMD, male sex, absence of urticaria pigmentosa, high levels of the bone resorption marker serum type I collagen C-telopeptide, and alcohol intake are known independent predictors of future fragility fractures [29,43]. The high prevalence of osteoporotic fractures supports the necessity of incorporating ISM into the differential diagnosis of idiopathic osteoporosis, particularly in female and male patients between the fourth and sixth decades of life [34,37,39].

## 3. Mechanisms Underlying Bone Impairment

Systemic mastocytosis is associated with certain bone remodeling abnormalities. Bone remodeling is a dynamic physiological process traditionally viewed as independent of immune cell involvement. However, increasing evidence highlights the immune system’s role in modulating bone metabolism under pathological conditions [44]. This emerging field has uncovered various cellular and molecular players that finely regulate the balance between bone-forming osteoblasts and bone-resorbing osteoclasts [45]. Central to this regulation are the receptor activator of nuclear factor kappa-B ligand (RANKL), the osteoprotegerin (OPG) signaling pathway, and the canonical WNT pathways, which are the primary signaling pathways that regulate bone remodeling and maintain skeletal homeostasis. These pathways promote osteoblast bone formation and osteoclast bone resorption accordingly [45,46].

RANKL is a molecule recognized as a signaling factor in bone, which also functions in the immune system [44]. RANKL is synthesized by osteoblasts and interacts with its receptor activator of nuclear factor kappa-B (RANK), present on osteoclasts. OPG, a naturally occurring RANKL antagonist that is also produced by osteoblasts, inhibits this mechanism [45]. RANKL, which is secreted by osteoblasts, facilitates and mediates the recruitment, activation, and survival of osteoclasts by binding to RANK, expressed by osteoclast precursors [41]. The secretion of RANKL is not limited to osteoblasts; it is also produced by BM stromal cells [41]. A decoy receptor for RANKL, OPG, is also expressed by these cells, making it a physiologic inhibitor of RANK–RANKL signaling [41]. Interestingly, the osteoblast is capable of expressing both the stimulator (RANKL) and the suppressor (OPG) of RANK, which in turn influences the activity and development of osteoclasts [41].

As MCs in SM can produce both RANKL and OPG, OPG and RANKL levels are elevated in patients with mastocytosis, which implies that the RANKL/RANK/OPG pathway is involved in bone changes associated with the disease [44,47]. OPG can interfere with the maturation of osteoclasts, hence diminishing bone resorption [45].

The differentiation of osteoblasts is primarily governed by the WNT/b-catenin signaling (canonical WNT pathway), which functions as the principal regulator of osteogenesis in conjunction with signaling molecules such as bone morphogenetic proteins (BMPs) [48]. Furthermore, the WNT pathway plays a key role in osteoblastogenesis, proliferation, and function of osteoblasts, and, in some circumstances, by promoting the osteoblast expression of OPG, it can lead to reduced osteoclastogenesis [49]. Additionally, the production of receptor inhibitors that bind to the WNT receptor, including sclerostin (SOST) and dickkopf-related protein 1 (DKK1), can modulate the regulation of the canonical WNT pathway in bone [50]. Furthermore, variations in the expression activity of the WNT pathway may account for a diverse array of bone mass phenotypes, including severe osteoporosis and sclerosteosis [50].

Sclerostin is a bone turnover protein with an important role in inhibiting the WNT signaling pathway, which is involved in osteoblast activation, proliferation, and differentiation. This results in reduced osteoblastic bone formation. Sclerostin also increases osteoclast formation by decreasing the expression of OPG. A study by Szudy-Szczyrek et al. showed that MCs are capable of sclerostin secretion and that after their stimulation by IL-6, a significant rise in the SOST gene expression occurs. This indicates that in mastocytosis-induced osteoporosis, MCs modulate not only the RANKL/RANK/OPG pathway but also the WNT pathway [30].

Additionally, a recent study identified MC-derived micro-RNA (miR-23a and miR-30a), which may be involved in the prevention of osteoblastogenesis and bone formation and warrants further research [51].

**Figure 2:**
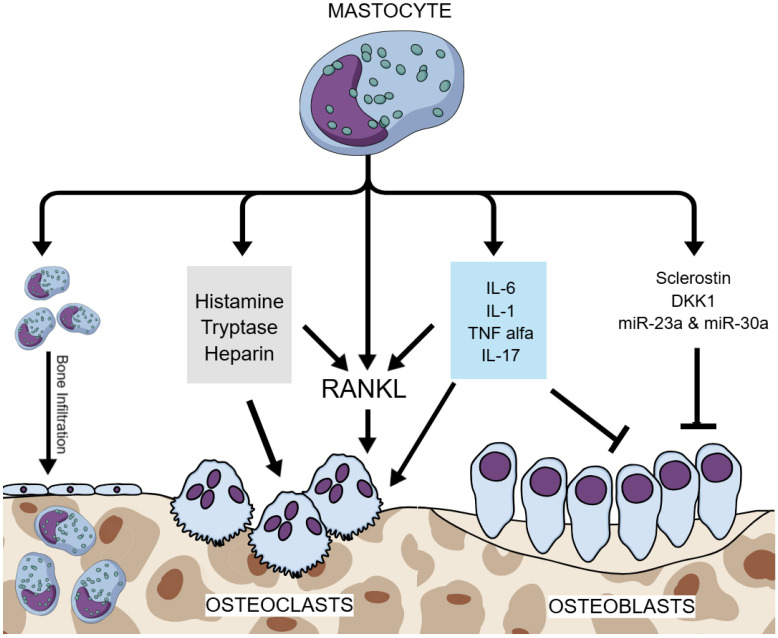
Pathophysiological mechanisms of ISM-induced osteoporosis. Legend: ISM—indolent systemic mastocytosis; RANKL—receptor activator of nuclear factor kappa-B ligand; IL-6—interleukin 6; IL-1—interleukin 1; IL-17—interleukin 17; TNF alpha—tumor necrosis factor alpha; DKK1—dickkopf-related protein 1; miR—microRNA; blunt arrow (┴)—signifies inhibition; pointed arrow (→)—signifies stimulation.

Several MC mediators are known to have a key role in bone metabolism. Neoplastic infiltration or the local release of mediators, such as histamine, heparin, tryptase, cytokines, and lipid mediators, has been suggested as the cause of skeletal alteration in SM [17]. Compared to healthy controls, patients with mastocytosis-induced bone density loss were shown to have higher levels of serum tryptase, IFN-γ, IL-1β, and IL-6 [32]. Tryptase induces the production of metalloproteases, which enhance bone resorption; cytokines, such as TNF-α, IL-1β, and IL-6, promote osteoclastogeneses and/or osteoclastic function; and histamine, prostaglandin, tryptase, heparin and platelet-activating factor activate the RANKL-induced pathway, all of which is known to promote osteoclastogenesis and, therefore, stimulate bone resorption [32]. Histamine metabolites have been shown to act on both osteoclasts and their precursors via autocrine and paracrine pathways, increasing osteoclastic activity and the risk of osteoporotic manifestation [52]. Figure 2 summarizes the pathophysiological mechanisms of ISM-induced osteoporosis.

The pathophysiology of SM-related osteosclerosis is ambiguous; nonetheless, it has been demonstrated that MCs can directly induce osteoblast proliferation, recruitment, and activity [41,53]. In contrast, MC-derived mediators, in particular tryptase, have been implicated in enhancing osteoblast function as well as in increasing OPG production, which reduces the differentiation and activity of osteoclasts. These results in suppression of osteoclast-mediated bone resorption and favor excessive bone accumulation [38,53].

Moreover, it is possible to infer that the number of MCs and their secreting activity may have opposing effects on bone turnover in this condition [41]. In contrast to patients with osteoporosis, when compared to healthy controls, patients with diffuse bone sclerosis showed significantly higher levels of serum tryptase, higher levels of biomarkers related to bone formation and turnover, low RANKL levels, lower levels of IFN-γ, and a trend toward lower IL-6 levels, indicating an immunosuppressive cytokine secretion profile [32].

## 4. Methods of Evaluation

### 4.1. Dual-Energy X-Ray Assessment

Dual-energy X-ray absorptiometry (DXA) is a crucial diagnostic tool for measuring BMD in patients with systemic mastocytosis (SM). It identifies osteoporosis as low BMD and helps manage the risk of fractures associated with SM, as low BMD is associated with increased fracture risk.

In patients with systemic mastocytosis, DXA scans are predominantly performed on the lumbar spine and proximal hip regions, providing important information about skeletal integrity. Osteoporosis is diagnosed when the T-score is ≤−2.5 SD [39,40]. DXA allows for precise classification of bone density status and helps clinicians differentiate between normal bone density, osteopenia, and osteoporosis, which is critical for determining subsequent treatment strategies [40,54].

DXA not only aids in diagnosis but also serves as a baseline for monitoring responses to therapies aimed at improving bone health. Following the initiation of antiresorptive treatment, such as zoledronic acid or denosumab, repeated DXA scans can inform clinicians about changes in BMD over time, allowing for therapy adjustments as needed [17,48,54].

Moreover, the relationship between elevated BST, indicative of mast cell activation, and reduced BMD has been explored in various studies. Research has indicated that patients with higher tryptase levels are likelier to exhibit lower BMD as measured by DXA, suggesting a direct impact of mast cell proliferation on bone density [48,55]. This correlation reinforces the importance of routine DXA assessments in monitoring patients with systemic mastocytosis, particularly those presenting with skeletal symptoms or unexplained fractures [39,48].

Longitudinal follow-up using DXA is also essential; studies have demonstrated distinct patterns in BMD changes among patients undergoing treatment for osteoporosis related to systemic mastocytosis, although, in one report, no decrease in BMD was registered in almost a two-year follow-up [56]. A cohort study indicated that patients receiving denosumab showed improvements in BMD at both lumbar and hip sites over one year, showcasing the effectiveness of this therapeutic strategy in managing mastocytosis-related osteoporosis [57]. Regular DXA assessment allows clinicians to track these therapeutic outcomes and stratify fracture risk more accurately over time.

### 4.2. X-Ray

X-ray imaging plays a vital role in evaluating bone involvement in patients with SM, particularly due to the condition’s associated complications, such as fractures. SM can lead to changes in bone structure, necessitating thorough radiologic assessment. Initial X-ray examinations can reveal osteosclerotic or osteolytic lesions. Bone involvement is frequently seen in the lumbar spine, where focal osteolytic lesions may be present. These appear as areas of decreased radiopacity, indicative of bone destruction. They are commonly found in the vertebral bodies of the lumbar spine and can significantly contribute to the risk of fractures [55]. Identifying these lesions via X-ray can prompt further investigation and management of the patient’s overall bone health [58]. Osteosclerosis is characterized by elevated radiopacity in areas of bone where mast cell infiltration has led to increased bone density. X-ray imaging can visually assess this, although advanced imaging techniques are often necessary to characterize these findings further [40,55]. Patients with SM may present with various types of fractures, particularly vertebral fractures, which can be detected through X-ray imaging [29,39]. The fractures identified can often be classified according to the Genant classification system, allowing for a standardized approach to diagnostic and treatment planning [39].

Vertebral fractures in SM can also be assessed with Vertebral Fracture Assessment (VFA), a method for detecting vertebral fractures by imaging the spine with DXA [59]. VFA is comparable to standard spine radiographs for detecting moderate (grade 2) and severe (grade 3) vertebral fractures. Additional benefits are a smaller dose of ionizing irradiation, greater patient convenience, and lower cost. If there is any doubt in the diagnosis of vertebral fracture, additional imaging is indicated [60].

Identifying significant osteolytic lesions or vertebral fractures can lead to interventions addressing the underlying mastocytosis and the resultant osteoporotic state. X-ray findings can guide treatment decisions, such as initiating bisphosphonate therapy or other targeted treatments, to improve bone density and prevent further fractures [29,40]. Regular follow-up imaging using X-rays can also help monitor disease progression. By comparing serial X-ray films, clinicians can assess changes in lesion characteristics or the emergence of new fractures, which may indicate worsening bone health or the disease’s progression and can assist in adapting treatment plans effectively [17].

### 4.3. Assessment of Microarchitecture and Bone Strength

Data on microarchitecture changes in cortical and cancellous bone among SM patients are conflicting. The methods for assessment include high-resolution peripheral quantitative computed tomography (HR-pQCT), magnetic resonance (MR), and histological evaluation of bone biopsies. Evaluation with HR-pQCT revealed higher volumetric BMD (vBMD), cortical vBMD, and cortical thickness at the radius in healthy controls compared to SM patients. However, those with a more advanced stage of SM show lower trabecular numbers and have an estimated failure to load at the tibia [61]. On the other hand, MR imaging did not reveal changes in SM and controls for trabecular number, trabecular thickness, and trabecular separations [62]. Bone histomorphometry and histopathological mechanisms revealed increased bone turnover with higher eroded area, osteoclast number, and bone formation rate compared to idiopathic osteoporosis. Regardless of osteoporosis or osteosclerosis phenotype, trabecular disorganization was noted. In this regard, more rod-like structures and poor connectivity were present, and compensatory structural changes with a higher number of trabeculae and less separation. Although higher trabecular bone volume was present in SM patients, large marrow spaces and perforated trabeculae were also observed in micro-CT images. SM patients had slightly thicker cortical bone, although not statistically significant (*p* = 0.10). Cortical bone formation was preserved, indicated by a comparable mineralizing apposition rate [35].

### 4.4. Bone Turnover Markers

Bone turnover markers (BTMs) provide insights into the dynamics of bone metabolism, reflecting the activity of osteoclasts and osteoblasts involved in bone formation and resorption processes. Research indicates that elevations in bone turnover markers are associated with a higher risk of osteoporosis and fractures in patients with systemic mastocytosis. Increased levels of these markers correlate with decreased BMD and are predictive of fragility fractures [39,54]. A study highlighted that elevated bone turnover markers are significant risk factors for osteoporosis in patients with SM, emphasizing the need for regular monitoring of these markers in managing bone health [56].

Correlations have been noted between BTMs and BST, reinforcing the connection between mast cell activity and bone health. Elevated tryptase levels have been linked to an increased risk of low BMD and osteoporosis, possibly due to the local release of osteoclast-activating factors by mast cells [17,54,55]. BTMs can be important adjunct measures to BMD assessments in evaluating skeletal health.

## 5. Constraints of Evaluation Methods

The evaluation of bone impairment in ISM presents several constraints, primarily due to disease heterogeneity, unique mechanisms of bone remodeling involved, and the limitations of conventional diagnostic tools.

Although BMD evaluation with DXA may aid in the diagnosis of osteopenia or osteoporosis in patients with ISM, its sensitivity and specificity to predict the risk of fractures are limited. This is likely due to altered bone microarchitecture and quality, mixed patterns of bone impairment, and the presence of focal lesions [17]. In fact, patients with mastocytosis may have fragility fractures even when the BMD is normal or slightly reduced [63], and fragility fractures are not only associated with osteoporosis but also with osteosclerosis or lytic lesions [64].

Focusing solely on BMD without conducting comprehensive evaluations can provide incomplete information about the clinical status of the bone and its metabolic pathology [28]. Additionally, the clinical symptoms of mastocytosis often overlap with those of other conditions, leading to potential misinterpretation and inaccurate diagnoses. For example, the osteoporotic symptoms associated with mastocytosis may be mistaken for primary osteoporosis, particularly in older individuals. To enhance the sensitivity of fracture risk assessments, employing a risk assessment tool such as MastFx is recommended. This tool considers clinical, laboratory, and BMD factors for a more thorough evaluation [26].

While X-rays provide valuable initial insights, they have some limitations, and sometimes there is a need for advanced imaging. These limitations include the inability to fully characterize the extent of osteosclerotic vs. osteolytic changes and the potential for subtle bone changes to go undetected. Consequently, complementary imaging techniques are often utilized, such as MRI for detailed soft tissue characterization and CT scans for enhanced bone visualization [40,57].

The assessment of bone quality and microarchitecture in ISM is crucial but also challenging. High-resolution peripheral quantitative computed tomography has been used, but its availability in clinical practice is limited [61].

BMTs provide insights into the systemic bone remodeling activity. However, there is an intrinsic limitation to the evaluation of focal bone lesions, and BMTs can only be indicative of generalized bone turnover increase and remodeling [17].

## 6. Therapeutic Approaches

Various treatment options are available to patients, contingent upon their clinical symptoms. In SM, treatment modalities encompass observation, augmented by preventative strategies to avoid MC degranulation, symptom management (such as addressing diarrhea), supportive interventions (osteoporosis management), and cytoreductive therapy aimed at MC reduction in cases of aggressive or treatment-resistant disease [16]. Early diagnosis of osteopenia prior to the onset of osteoporosis is the most critical objective in its treatment [22]. Calcium absorption may be impaired by malabsorption, and sodium cromolyn therapy may be beneficial for enhancing intestinal absorption [22]. Additionally, treating osteopenia may involve calcium, Vitamin D, and estrogen replacement in postmenopausal women [22].

The treatment of osteoporosis caused by ISM is primarily based on antiresorptive medications [65]. Despite the fact that anti-fracture efficacy has not yet been confirmed by long-term randomized studies, the current evidence indicates that bisphosphonates are the primary treatment for mastocytosis-related osteoporosis [17]. In their study, Barete et al. observed that in ISM patients, bisphosphonate treatment resulted in a stable mean hip BMD, a substantial increase in lumbar BMD with an average increase of 2% per year, and no new fractures over the 65-month follow-up [40]. Notably, in a retrospective analysis, Lim et al. reported that patients indicated a reduction in bone pain following bisphosphonate therapy [65]. However, because of the aggravation of digestive symptoms and the possibility of severe acute-phase reaction following intravenous application, these medications are not always preferred in SM [17,37]. Acute-phase reactions can be effectively reduced by paracetamol 0.65–1.0 g administered every 6 h for three days, dexamethasone (4 mg daily for three–seven days), methylprednisolone (40 mg for two days), and ibuprofen [66]. Additionally, the residual long-term effect of bisphosphonates is a cause for concern, particularly in women who are of reproductive age [17].

The results of a study conducted by Rossini et al. on 25 patients with ISM who were treated with a single 5-mg intravenous infusion of aminobisphosphonate zoledronate were recently reported [67]. A single intravenous infusion of 5 mg of zoledronic acid given to patients with osteoporosis secondary to ISM is associated with decreases in bone turnover markers and also substantial increases in BMD of the spine and hip for a minimum of one year [48]. Nevertheless, a prevalent side effect is the acute phase reaction following the initial administration; this is a temporary response that can be mitigated through prior medication and thorough patient education [48].

Denosumab, a biologic anti-resorptive drug, has been developed in recent years to treat postmenopausal osteoporosis [17]. In order to disrupt the osteoclast’s RANK-RANKL activation pathway, this medication employs monoclonal antibodies against RANKL [17]. Despite the lack of clinical data and certain safety concerns regarding the risk of allergy, denosumab may be a particularly good substitute in cases of bisphosphonate intolerance [17]. Orsolini et al. reported that after 12 months of treatment with this medication, BMD increased at lumbar and femoral sites, in contrast with serum tryptase levels, bALP, and C-terminal telopeptide of type I collagen (CTX) concentrations, which decreased; additionally, no new vertebral fractures were detected [57].

The stimulation of osteoblasts with teriparatide, the 1–34 active fragment of parathyroid hormone, was proposed, particularly in the presence of low bone formation markers [17]. However, the description of a rise in MCs in parathyroid bone disease raises some safety concerns in SM [68]. Further investigation is required. Nevertheless, since teriparatide may promote the growth and proliferation of atypical MCs and cause more aggressive types of SM, it is advised not to be suggested as an alternate treatment for mastocytosis-related osteoporosis [17].

In cases of severe osteoporosis, it is beneficial to incorporate cytoreductive agents, such as tyrosine kinase inhibitors (TKI), as they target KIT and provide enhanced benefits when used in conjunction with bisphosphonates [17]. Furthermore, low-to-intermediate doses of interferon alpha (IFN-α) (i.e., 1.5 to 3 million units three times a week) have been shown to produce positive results in a small number of cases of severe osteoporosis in patients at risk of developing vertebral collapse or other pathological fractures [22]. To the best of our knowledge, there is no data on BMD and bone fractures in SM patients treated only with IFN-α; however, one study shows a greater increase of BMD in SM patients treated with IFN-α and pamidronate compared to pamidronate alone (16.05+/−6.12% vs. +0.2+/−2.13% at the spine, 5+/−2.24% vs. −2.25+/−2.78% at the femoral neck) [69]. Although its beneficial effects are limited by substantial toxicities, it has been demonstrated to alleviate symptoms associated with MC mediators, decrease BM MC infiltration, and reduce mastocytosis-related osteoporosis [70]. However, prior to initiation of IFN-α therapy, it is crucial to conduct a comprehensive assessment of potential adverse effects and the presence of other pathological conditions that render IFN-α administration contraindicated, including ischemic cardiopathy, severe hepatopathy, and depression [22].

Moreover, in cases requiring accelerated cytoreduction or in instances of demonstrated intolerance to IFN-α, it is advised to change the treatment to 2-chlorodeoxyadenosine (cladribine or 2-CdA) 37 [70]. Cladribine, compared to other TKIs, has the potential to provide time-limited therapy with three–six cycles and has a well-understood side effect profile [16]. The potential toxicities of 2-CdA encompass myelosuppression and lymphopenia, which elevate the risk of opportunistic infections [70]. Furthermore, using appropriate prophylactic antibiotic/antiviral therapies can reduce this risk [16]. During treatment with cladribine, improvements in bone-related symptoms such as reduced fractures were reported. However, these outcomes were not explicitly tied to BMD. The probable mechanism is through the MC burden and the osteolytic mediators that they release [71].

Additionally, Midostaurin (PKC412) is a multitargeted TKI that received approval from the U.S. Food and Drug Administration (FDA) for the treatment of adult patients with AdvSM in 2017. It has been shown to significantly decrease the BST and BM MCs burden in 40% to 50% of cases [72]. Furthermore, in patients who have not improved following IFN-α or cladribine therapy, this medication is a suitable salvage treatment [16]. Administering Midostaurin can be problematic due to the prevalence of gastrointestinal adverse effects [16]. Additionally, during treatment, patients require monitoring of amylase/lipase levels, liver function tests, blood counts, and ECG (QTc interval) [16]. If demonstrated to be generally safe for prolonged usage, Midostaurin may play a role in the management of ISM and SSM [70]. In a case report of a 69-year-old female with SM treated with denosumab and Midostaurin, a rapid increase in BMD was observed, which was the most probable consequence of pro-osteoblastic mediators released by abnormal mast cells, as tryptase levels increased amid treatment [73]. A single-center study on 37 osteoporotic patients with SM failed to demonstrate the benefits of Midostaurin compared to combined IFN-α and pamidronate treatment [74].

In 2021, the FDA approved Avapritinib as a first-line therapy for adult patients with AdvSM [16]. It specifically targets activation-loop mutations of *KIT*, notably *KIT* D816V [16]. Initial studies are encouraging, demonstrating potential disease-modifying effects and clinically significant responses linked to biochemical and histopathological outcomes [16]. Furthermore, during the double-blind phase of an ongoing randomized trial in patients with ISM (PIONEER), Avapritinib demonstrated statistically significant enhancements in ISM symptoms and reductions in objective indicators of MCs burden, including BST, BM MCs, and *KIT* D816V variant allele frequency (VAF), following 24 weeks of treatment [75]. However, the tolerance of long-term treatment is still a concern due to adverse effects on cognition (e.g., confusion, dizziness, and memory impairment) and the emergence of intracranial bleeding [16].

There are a few medications being investigated. Bezuclastinib is an oral, powerful TKI that has low permeability across the blood–brain barrier. It is undergoing investigation in two trials: the APEX and the SUMMIT studies. The APEX study’s recent results showed that all 11 patients exhibited a reduction of over 50% in BST; following a minimum of two treatment cycles, eight patients exhibited a reduction of at least 50% in MB MCs load, with six attaining total MC aggregates [16]. Another drug being tested in the AZURE and HARBOR studies is BLU-263, a selective KITD816V inhibitor, distinct from Avapritinib due to its restricted brain penetration capability [16]. Additionally, in a randomized, double-blind, placebo-controlled study, another TKI, Masitinib, has shown a small improvement after 24 weeks, with an 18.7% cumulative response (symptom control) compared to 7.4% for the placebo [76]. Additionally, there were no life-threatening toxicities, and mean tryptase levels decreased [76].

The management of ISM is a multifaceted approach tailored to individual patient needs and clinical presentations. Emerging therapies, including targeted TKIs, offer potential benefits but also come with risks that necessitate careful monitoring and patient education. Ongoing research into novel treatments continues to expand the therapeutic landscape, providing hope for improved outcomes. Table 1 summarizes the pharmacological management of SM-induced osteoporosis.

## 7. Conclusions

The identification of bone involvement in patients with SM is essential due to the heterogeneity of manifestations and intervention strategies. It is recommended that SM be considered as a potential diagnosis in the screening of all premenopausal women and men who present with an unexplained fragility fracture or low BMD, as well as postmenopausal women who have a suspicion of secondary osteoporosis [17]. Furthermore, in patients with a history of severe anaphylaxis or other clinical symptoms associated with the release of MC mediators, mastocytosis should be suspected [17,79].

An early identification of osteoporosis resulting from mastocytosis facilitates the commencement of suitable pharmaceutical intervention. The literature indicates multiple viable therapy approaches that can enhance BMD and decrease fracture risk, therefore enhancing the patient’s quality of life [48]. Furthermore, the potential for a decreased risk of CNS adverse events may be achieved through the development of a new generation of KIT-targeting TKI medications with minimal CSF penetration [16]. Additionally, the collected data support the investigation of RANKL, OPG, and SOST as potential diagnostic markers for osteoporosis related to SM: these cytokines may serve as novel treatment targets in mastocytosis with bone implications [45].

Given that this is a rare disease necessitating a multidisciplinary approach, it is important for patients to be monitored [48]. Ultimately, gaining a more comprehensive understanding of this disease may facilitate the diagnosis and the selection of an individualized treatment plan, optimizing patient care and addressing the complexities of this condition [24].

## Figures and Tables

**Table 1:** Pharmacological management of SM-induced osteoporosis.

Drug/Treatment	Mechanism	Therapeutic Effects/Benefits	Adverse Effects/Limitations
Bisphosphonates (aminobisphosphonate Zoledronate) [17,37,40,65]	Antiresorptive agents	↑ lumbar and hip BMD (~2%/year); ↓ bone turnover markers; ↓ bone pain; no new fractures observed	GI symptoms (oral bisphosphonates); acute phase reaction after 1st dose (usually temporary, can be pre-medicated); caution in reproductive-age women; atypical femoral fracture; osteonecrosis of the jaw; hypocalcemia;
Denosumab [17,57]	Biologic anti-resorptive drug (anti-RANKL monoclonal antibody)	↑ BMD (lumbar, femoral); ↓ serum tryptase, bALP, CTX; no new vertebral fractures; (short term—12 months, small cohort N = 4); useful in bisphosphonate intolerance	Allergy risk; lacks long-term data in SM; rebound fractures (in case of delay/discontinuation after >1 application); atypical femoral fracture; osteonecrosis of the jaw; hypocalcemia;
Teriparatide [17,68]	Recombinant PTH	Stimulates osteoblasts (theoretical benefit)	May ↑ atypical MC proliferation; potential for disease worsening; not recommended in SM
IFN-α [22,70]	TKI	↓ MC burden; ↓ MC mediator symptoms; improved BMD in severe cases; ↓ BM MC infiltration; reduced mastocytosis-related osteoporosis	Toxicities: flu-like symptoms, depression, hepatic/cardiac contraindications
Cladribine (2-CdA) [16,70]	TKI	↓ MC burden; potential for disease control in severe cases	Myelosuppression, lymphopenia, infection risk (needs prophylaxis)
Midostaurin (PKC412) [16,70,72]	TKI	↓ serum tryptase, ↓ BM MC burden; salvage therapy option	GI side effects; requires monitoring (for labs and ECG); long-term safety under investigation
Avapritinib [16,75]	TKI	↓ tryptase, ↓ KIT VAF, ↓ BM MCs; ↑ symptom control	CNS side effects (confusion, dizziness); intracranial bleeding risk
Bezuclastinib [16,77]	TKI	↓ tryptase (>50% in all treated); ↓ BM MC burden; some complete MC clearance in early trials	Low CNS penetration; safety profile still emerging
BLU-263 [16,78]	TKI	Similar target to Avapritinib; designed to limit CNS penetration	Limited CNS penetration; long-term data pending
Masitinib [76]	TKI	Symptom improvement; ↓ mean tryptase	Mild side effects; no life-threatening toxicities
Supportive Therapies [22]	Calcium, vitamin D, estrogen	Treats osteopenia/osteoporosis; improves calcium balance; sodium cromolyn improves absorption	Malabsorption can limit efficacy; estrogen contraindicated in some populations

Legend: BMD—bone mineral density; GI—gastrointestinal; RANKL—receptor activator of nuclear factor kappa-B ligand; bALP—bone-specific alkaline phosphatase; CTX—C-terminal telopeptide of type I collagen; MC—mast cell; SM—advanced systemic mastocytosis; ISM—indolent systemic mastocytosis; TKI—tyrosine kinase inhibitors; IFN-α—interferon alpha; Cladribine or 2-CdA—2-chlorodeoxyadenosine; VAF—variant allele frequency; CNS—central nervous system; ↑—signifies an increase; ↓—signifies a decrease.

## Data Availability

No new data were created or analyzed in this study. Data sharing is not applicable to this article.
